# An Analysis of Anxiety-Related Postings on Sina Weibo

**DOI:** 10.3390/ijerph14070775

**Published:** 2017-07-13

**Authors:** Xianyun Tian, Fang He, Philip Batterham, Zheng Wang, Guang Yu

**Affiliations:** 1 School of Management, Harbin Institute of Technology, Harbin 150001, China; uncertainorcertain@gmail.com (X.T.); hefang@hit.edu.cn (F.H.); wzxdmail@gmail.com (Z.W.); 2 Centre for Mental Health Research, The Australian National University, Canberra, ACT 2601, Australia; philip.batterham@anu.edu.au

**Keywords:** social media, mental health, public health, anxiety, Sina Weibo

## Abstract

This study examines anxiety-related postings on Sina Weibo to gain insight into social networking about mental health. The themes of a random sample of anxiety-related postings (*n* = 1000) were assessed. The disclosure of anxiety was the most common theme. The prevalence of anxiety was higher in certain areas where the economy is stronger than others, and the people living there suffered from more stress. Users who talked about feeling anxious tended to be more active on social media during leisure hours and less active during work hours. Our findings may be developed to detect and help individuals who may suffer from anxiety disorders at a low cost.

## 1. Introduction

Like Twitter and Facebook, Weibo is a social media site for communication and information sharing in China. As the most influential social media site in China, Sina Weibo has attracted more than 500 million users. In other words, it may directly influence about one-third of China’s population. Weibo allows its users to post updates that are less than 140 Chinese characters. These postings can be reviewed by people who follow them.

More than 100 million postings are posted on Weibo every day. These postings cover many different topics, from news, product promotions, and document sharing to comments on social events. The variety and richness make Weibo an excellent platform for companies to build their brands and for the government to understand the population’s needs and difficulties better. In addition, Weibo is also a valuable data source for various types of research. Because of its anonymity, people can talk about private issues freely on Weibo, such as their physical and mental health concerns [1,2,3,4,5].

Anxiety disorders are a category of mental disorders characterized by feelings of anxiety and fear, where anxiety is a worry about future events, and fear is a reaction to current events [6]. It is estimated that 18% of American adults and 14% of Europeans may suffer from one or more anxiety disorders [7,8]. There are many types of anxiety disorders, including generalized anxiety disorder, social phobia, specific phobias, panic disorder, and agoraphobia. Social anxiety disorder [9] is the most common anxiety disorder and one of the most common mental illnesses, with about 12% of American adults having experienced it [9,10]. If people who are bothered by social anxiety disorder are undiagnosed or untreated, they may develop some other disorders, such as substance abuse disorders and depression.

Despite the prevalence and severe consequences of anxiety disorders, the rate of underdetected and undertreated disorder is substantial [11,12,13]. One resolution to this problem may be to deliver tailored information to people with anxiety who are reluctant to seek professional help. A tailored program could also be used to reduce stigma, an important barrier to help seeking, so that more people are encouraged to seek professional help. Social media might be a useful tool to deliver such information, due to its extensive use and low cost.

Recent studies have shown the feasibility of using social media to investigate various health-related issues. For instance, researchers have explored sleep complaints on Sina Weibo to gain insights into social networking about mental health. Their results suggest that the Sina Weibo can be used as a tool to identify people experiencing insomnia [4]. The potential of using Twitter to detect and diagnose major depressive disorder and suicide risk in individuals has also been explored [14,15]. In addition, the association of between online detection and offline depression diagnosis has also been established [16], indicating that individuals who displayed one or more text references to depression symptoms on publicly available Facebook profiles had higher Patient Health Questionnaire-9 scores. However, research to explore the potential of using social media to investigate anxiety-related issues is in its infancy.

To date, studies have mainly focused on analyzing the association between social anxiety disorder and social media use. For instance, Fernadez et al. had amateur coders rate levels of social anxiety based on Facebook profiles, and tested the association between the rating and the participants’ self-reported social anxiety symptoms [17]. Their results suggest that social anxiety is recognizable both in objective criteria by viewing Facebook profile and from raters’ impressions of the Facebook profile. However, no studies have examined other kinds of anxiety disorders, such as generalized anxiety disorder and phobias. In additional, although the feasibility of using social media to predict social anxiety has been validated [17], social media use and demographic characteristics of individuals who may suffer social anxiety have not been well studied.

This study was designed to investigate whether and how anxiety disorders are discussed online and to explore the potential of social media for identifying and informing the prevention of the anxiety disorders. Specifically, this study aims to understand: (1) whether references to anxiety disorders can be extracted from social media; (2) the themes of anxiety-related postings; (3) the characteristics of individuals who may be experiencing an anxiety disorder.

## 2. Method

### 2.1. Data Collection

From 17 May to 21 August 2015, approximately 394 million postings were obtained from Sina Weibo through its Application Programming Interface (API). These postings belonged to one million users who were also randomly sampled from Sina Weibo. For every user in this sample, his/her latest 1000 postings were collected. If the number of his/her all postings was fewer than 1000, then all of his/her postings were sampled. Demographic characteristics and content of the posting were collected for analysis.

**Figure 1:**
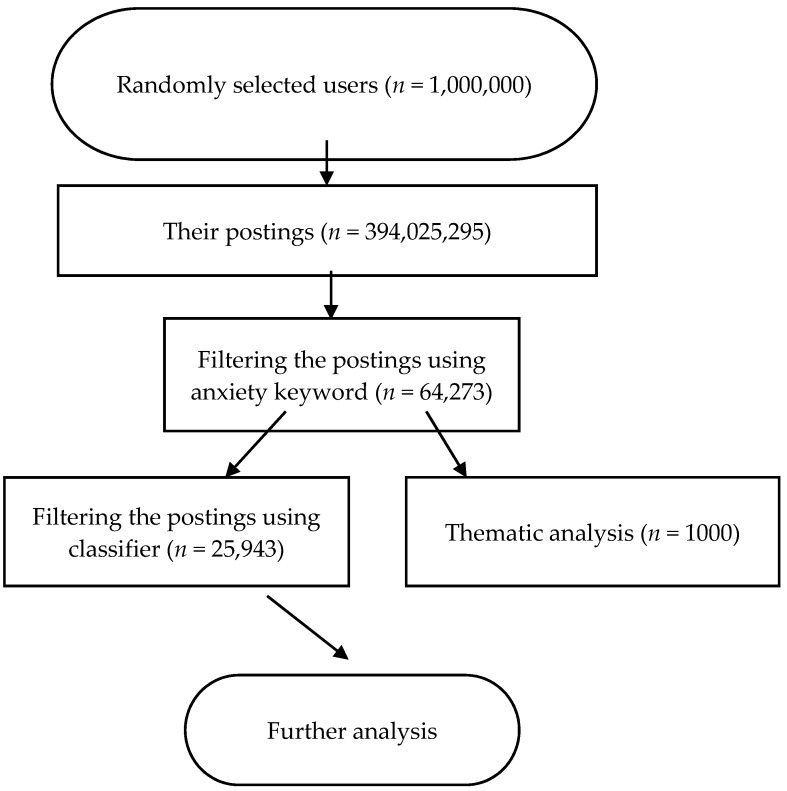
Flowchart of postings related to anxiety.

The data used in the current study is publically accessible. However, to protect the users’ privacy, any information that could be used to trace the user was stripped from the data set. The flow chart of the data processing is presented in Figure 1.

### 2.2. Themes

All postings that contained the keyword “焦虑” (anxiety) were retrieved from the 394,025,295 postings. The number of the selected anxiety-related postings was 64,273 (i.e., 0.02%). The reason for not including other anxiety-related words, such as “害怕” (fear), “担心” (worry), and “恐怖” (phobia), was that these words were not anxiety-specific enough, and including them would introduce too much noise.

Of the 64,273 postings initially selected through the keyword-based method, 1000 postings were randomly sampled to identify themes contained in postings that contained the keyword “anxiety”. Two researchers experienced in mental health reviewed the 1000 postings to determine the most common themes in the postings. Themes were defined as topics that occur and reoccur [18]. A codebook was created after the review which was used to determine the proportions of different themes. Postings were coded as follows: (1) users disclosing their anxieties; (2) philosophical thoughts on anxiety; (3) how users cope with their anxieties; (4) help seeking; (5) sharing medical information on anxiety.

A three step coding process was employed to code the postings. Firstly, two research members coded the 1000 postings using the codebook. Secondly, they discussed each of the postings where there was a disagreement until agreement was reached. Finally, a third research member who was also experienced in mental health was introduced to code 100 postings which were randomly sampled from the 1000 postings, to compute the inter-rater reliability. The inter-rater reliability for each theme was as follows: (1) users disclosing their anxieties: agreement 83%, kappa 0.625; (2) philosophical thoughts on anxiety: agreement 94%, kappa 0.819; (3) how users cope with their anxieties: agreement 92%, kappa 0.51; (4) help seeking: agreement 96%, kappa 0.757; (5) sharing medical information on anxiety: agreement 94%, kappa 0.766.

### 2.3. Selection of Postings

As mentioned above, not all postings that contain the keyword “anxiety” refer to the disclosure of anxiety. Hence, a text classifier was required to detect postings indicating that the people who posted them may suffer from anxiety. A text classifier is one kind of model which is widely used in text classification tasks. In this study, it was employed to distinguish the disclosure of anxiety from other kinds of postings.

In this paper, we consider detection as a binary classification problem. For the supervised classification problem, a training data set is required. However, as far as we know, there is no such data set available. Thus, 5000 postings were randomly selected from the 64,273 postings. Our three researchers with mental health experiences independently labelled the postings, and majority rule was applied when there was no agreement. Three kinds of machine learning algorithms—including Logistic Regression, Random Forest, and Support Vector Machine (SVM) [19]—were employed to build the classifiers. Ten-folded cross-validation and accuracy were selected as measures to evaluate the performance of the classifiers.

Experimental results suggested that among the classifiers, SVM achieved the highest accuracy with 89%. Consequently, the SVM classifier was employed by us to classify the 64,273 postings to identify the target postings. In the end, 25,943 postings were identified as reflecting disclosure of anxiety and consequently included for further analysis.

### 2.4. Demographic Characteristics

To investigate the demographic characteristics of individuals who may suffer from anxiety, we examined the positive postings classified by the text classifier mentioned above. The individuals who posted more than two positive postings were the target population for this analysis as they may suffer from more severe anxiety; 3111 users were found to meet this requirement. Their demographic and usage characteristics—including name, gender, location of the individual, number of followers, number of messages, number of followings, description, and type of user—were extracted using the API offered by Sina Weibo.

A comparison group was sampled by selecting individuals who shared the same activity level with the users who displayed anxiety. The “activity level” was defined as the ratio of the number of postings to the number of the days from every account’s creation date to the date when we crawled the data. The comparison group also comprised 3111 users. Pearson Chi-square tests were performed to compare the characteristics of anxious users and typical users (users from the comparison group) based on gender. Wilcoxon Rank Sum and Signed Rank Test [20] were used to determine whether there were significant differences between anxious users and typical users on the basis of the number of followers, number of following, and number of postings. *p* < 0.05 was considered statistically significant. Cohen’s d was used to calculate the effect sizes between anxiety displayers and typical users on number of following, number of followers, and number of postings.

To examine the geographic distribution of anxious users, we divided the number of the anxious users per province (or autonomous region, municipality, or special administrative region) by the population in that area.

### 2.5. Diurnal Pattern

To explore the diurnal pattern of the postings posted by people who may suffer from anxiety, we retrieved all of their postings from the database. The number of these postings was 2,399,206. The postings posted by the comparison group of typical users were also retrieved from the database. The number of these postings was 2,116,085. We calculated the number of the postings that were made per hour by anxious users and typical users.

Each day was divided into three periods: sleep hours, work hours, and leisure hours. The number of postings posted by anxious users and typical users in each period was calculated. Pearson Chi-square was used to determine whether the difference of distribution of posting in these three periods between the two groups was statistically significant. *p* < 0.05 was considered statistically significant.

### 2.6. Relationship with Depression and Insomnia

Previous studies have shown that the people who suffer from anxiety may also be bothered by depression and insomnia, so we attempted to determine whether an association between anxiety and insomnia could be found using the social media data. We collected all the postings posted by the anxious users and counted the number of the postings that contained the keyword “抑郁” (depression), “睡不着” (can’t fall asleep) or “失眠” (insomnia). For comparison purposes, we calculated the proportions of the comparison group’s postings that contained the same keywords. Pearson Chi-square tests were employed to determine whether the difference between users displaying anxiety and typical users on the frequency of specific keywords is statistically significant. *p* < 0.05 was considered statistically significant.

## 3. Results

### 3.1. Themes

We randomly selected 1000 postings as the sample of the population of postings that contained the keyword anxiety. Of the 1000 postings, 103 (10.3%) were excluded because they were advertisements or ambiguous. Themes identified in the 897 postings are shown in Table 1.

The most common theme of the postings (*n* = 328, 36.6%) was disclosure of anxiety. As we mentioned before, anxiety disorders include many different types, but people just disclosed that they were anxious rather than specifying a specific kind of anxiety disorder. The second most popular theme (*n* = 262, 29.2%) was philosophical thoughts on anxiety. These thoughts generally had the intention of helping people to prevent or reduce their anxiety in a non-professional way. About 19% (*n* = 168) of the postings were shared medical information on anxiety from a psychological or physical perspective. The other themes were much less frequent. Only 7% (*n* = 59) of the postings attempted to seek help from others. About 9% (*n* = 80) of the postings mentioned some measures were taken to prevent or reduce anxiety. However, not all of these actions may have been useful. For example, some users attempted to reduce their anxiety by talking nonsense or smoking.

### 3.2. Demographic Characteristics of Users

The analysis of gender distribution illustrates that females tended to disclose anxiety more frequently than males (Table 2). The results also suggest that anxious users followed fewer users than typical users. The differences between two groups on postings and followers were not statistically significant.

**Figure 2:**
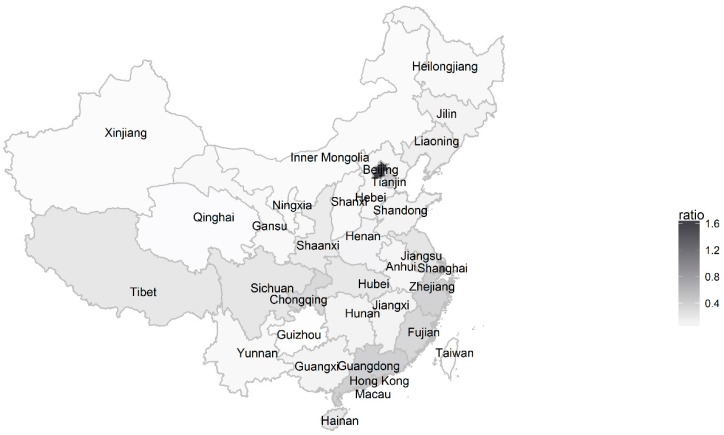
Geographic distribution of users with anxiety.

The geographic distribution of the users displaying anxiety is presented in Figure 2. The ratio is the number of the anxiety displayers per 100,000 population per area. Figure 2 shows that the ratio was higher in the southeast region of China, including the Zhejiang, Fujian, and Guangdong. These three provinces are three of the richest provinces in China. In addition, some big cities, such as Beijing and Shanghai, also had more anxious users than other areas. These two places are the two largest cities in China.

### 3.3. Diurnal Activity

**Figure 3:**
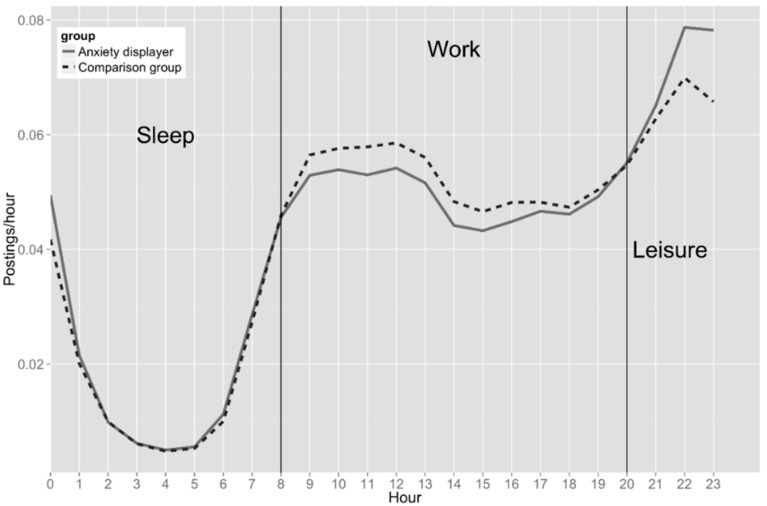
Diurnal trends (average number of postings posted hourly through a day).

The difference between two groups on distribution of postings was statistically significant. Figure 3 presents the diurnal pattern of the posted messages by the users displaying anxiety and comparison users, indicating that the general population was more active during work time than users with anxiety. In contrast, the anxiety displayers were more active during leisure time. There was only a slight difference in activity level between the two groups during sleep time.

### 3.4. Relationship with Depression and Insomnia

**Figure 4:**
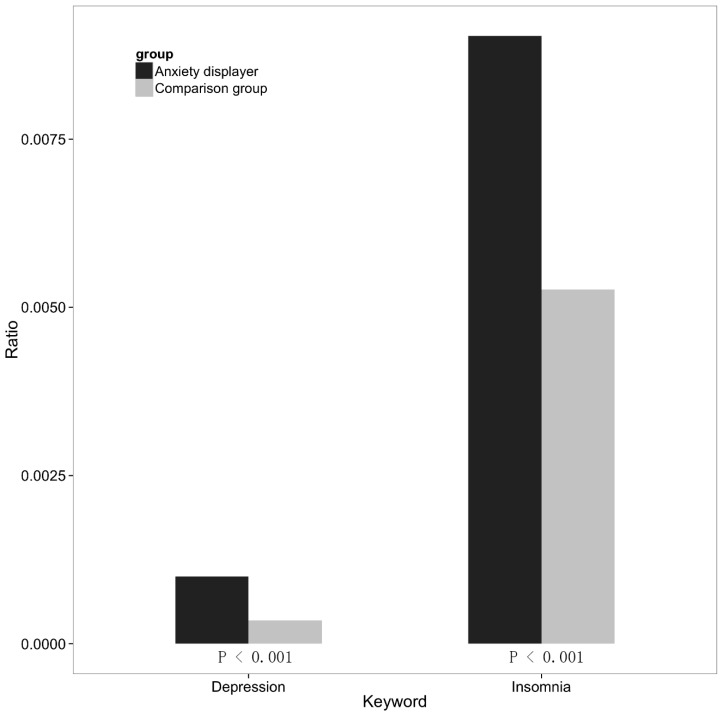
Relationship with depression and insomnia.

Figure 4 shows the proportions of the postings that contain the depression-related keyword and insomnia-related keyword in the anxiety displayers and the typical users. Chi-square test indicates that the differences between users displaying anxiety and typical users on the depression-specific keywords and insomnia-specific keywords were both statistically significant. From this figure, we can observe that users who disclosed anxiety mentioned both insomnia and depression more frequently in their social media postings than typical users. The pattern shown here is consistent with the previous studies indicating that the people who suffer from anxiety may also experience insomnia and anxiety. The consistency of these results supports the effectiveness of the method we used to identify users with anxiety. Additionally, this method may be utilized as a complementary approach to determine the individuals at increased risk of insomnia and depression.

## 4. Discussion

This study investigated anxiety-related postings on Sina Weibo to examine characteristics of individuals who mentioned anxiety, what aspects of anxiety were discussed, and how people coped with anxiety. In this study, 3111 users who disclosed anxiety more than twice were identified. We conducted a thematic analysis of the anxiety-related postings and found that the most common theme was the disclosure of anxiety. The proportions of different themes suggest that users displaying anxiety can express their symptoms but may not know how to seek professional help to overcome their anxiety. What they tend to rely on is the consolation of philosophy. The reason why they rely on philosophy may be that most of users do not consider anxiety as a potentially serious mental health issue. More research is required to determine the degree to which the individuals identified using this methodology meet clinical criteria for an anxiety disorder. However, we consider this approach to be a valuable complement to the traditional clinical approach, as it may help to identify individuals who may suffer from anxiety but do not typically seek help from professional service setting. This may lead to the development of low cost interventions to provide tailored health information online.

The demographic characteristics of users who disclosed anxiety and may suffer from anxiety were also evaluated in this study. The gender distribution result was consistent with previous research indicating that women have a higher prevalence of anxiety disorders than men. This gender difference implies that more attention should be paid to females when delivering tailored anxiety information to users, although it may also be the case that males are more reluctant to disclose anxiety symptoms. Only one previous study has described the demographic features of people who may suffer from social anxiety on social media [17]. Thus, as far as we know, this is the first study that focuses on all forms of anxiety, and not only on social anxiety. In addition, this is the first study which assesses the prevalence of anxiety in users of Chinese social media.

The geographic pattern of users with anxiety indicated that the prevalence of anxiety was higher in certain areas than others. A possible reason that individuals who lived in rich areas including big cities tended to express anxiety may be that people living in these areas experience more frequent stressors such as competition and social fragmentation. Another explanation may be that people living in these areas are more knowledgeable about anxiety, resulting in more disclosure of anxiety. Our discovery on the geographic distribution of anxious users may be helpful in assisting the Chinese government to distribute health resources efficiently, given its large population and limited resources.

We also found that people who may suffer from anxiety tended to be more active on social media during leisure time. More research is required to explain why the pattern is like this. However, one possible reason may be that anxious users experience more workplace stress so that they are less able to post during work time, preferring instead to use social media during leisure time. It may consequently be more efficient to deliver tailored information to these persons during leisure hours when they are more active on social media.

We also found that users who disclosed anxiety had more postings about depression and insomnia. This pattern suggests that these individuals may also be bothered by insomnia and depression, consistent with previous studies demonstrating high comorbidity between anxiety, depression, and insomnia [21,22]. The consistency of these results supports the effectiveness of the method we used to identify users with anxiety. Additionally, it supports the current method as a complementary approach to identify individuals at increased risk of insomnia and depression. The finding of elevated depression and insomnia in users reporting anxiety suggests a need for close attention to the design of treatment and prevention programs for anxiety, as they may need to account for this comorbidity.

In this study, we could not precisely infer the specific type of anxiety disorder that an individual may suffer from. The majority of postings posted on Sina Weibo consist of only one or two sentences limiting our ability to distinguish between different manifestations of anxiety from a single posting. In addition, most of the users did not demonstrate professional knowledge about anxiety, which means that their self-diagnosis may be unreliable. More research is required to establish the association between specific symptoms and users’ activities on social media. Demographic features of anxious users were also not fully described. For example, data on age distribution was not available.

It takes more resources and time to actively find individuals who may suffer from anxiety disorders using traditional epidemiological or clinical methods. In contrast, the method used in this study offers researchers and health services a great opportunity to identify and assist these people at a low cost. For example, tailored programs could be delivered to individuals who disclose anxiety frequently or seek help on social media. Such programs could help these individuals understand more about anxiety disorders and encourage some of them to seek professional help, although more research is required on how best to implement such programs. It is worth noting that anxiety symptoms are common and normal in the general population and this does not mean that all these people have undiagnosed anxiety disorders. Because there are some people who are high on neuroticism [23,24] that will regularly report feelings of anxiety, but are still functioning and “healthy”.

Although social media provides researchers with a great opportunity to collect data that would otherwise require significant resources, it also brings new challenges that traditional ethics frameworks are not equipped to deal with. Firstly, it is unclear whether publicly-available data should be used to identify individuals at risk of a health condition and how results of the data analysis can be appropriately used for public health applications. Secondly, it is also unclear how identified individuals could be appropriately assisted without invading their privacy. More research is required to establish a new framework to guide researchers and public health officials in this field.

## 5. Conclusions

Despite these limitations, our study offers a unique understanding of how social media is used in discussing anxiety. We found that many users on Sina Weibo mentioned anxiety and some of them were likely to suffer from anxiety. The most popular theme was the disclosure of anxiety. In addition, a keyword analysis was conducted to estimate the prevalence of depression and insomnia among users of social media reporting anxiety. This study also shed light on demographic features and diurnal patterns of individuals who may suffer from anxiety. Our findings may help health professionals to better understand patterns of anxiety in the community. Our findings may inform the development of more efficient programs to prevent and treat anxiety in the community.

## Figures and Tables

**Table 1:** Themes of anxiety-related postings.

Themes	*n* (%)	Example Postings
Disclosure of anxiety	328 (36.6%)	I am suffering from anxiety, so crazy.
Philosophical thoughts on anxiety	262 (29.2%)	For most people, the anxiety rises from the unfair expectation; maybe we should choose a different way of living.
How do users cope with anxiety	80 (8.9%)	I am talking nonsense to reduce my anxiety.
Help seeking	59 (6.6%)	What kind of happiness can help me to forget the anxiety?
Shared medical information	168 (18.7%)	#Sohu Health# The change of the pregnancy hormones can lead to anxiety, it is inevitable, but not severe.

**Table 2:** Demographic characteristics of users.

Demographic Variables	Disclosed Anxiety	Comparison Group	Effect Size	*p*
Gender				
Male	19.7%	39.7%		<0.001
Female	80.3%	60.3%		
Following				
Quartile 1	92	105	0.181	<0.001
Median	165	182		
Quartile 3	273	309.5		
Followers				
Quartile 1	104	94	0.014	0.909
Median	175	170		
Quartile 3	291	303.5		
Postings				
Quartile 1	451	423	0.005	0.057
Median	741	703		
Quartile 3	1196	1164		
